# Carbonic anhydrases in development: morphological observations and gene expression profiling in sea urchin embryos exposed to acetazolamide

**DOI:** 10.1098/rsob.220254

**Published:** 2023-01-04

**Authors:** Francesca Zito, Rosa Bonaventura, Caterina Costa, Roberta Russo

**Affiliations:** Istituto per la Ricerca e l'Innovazione Biomedica, Consiglio Nazionale delle Ricerche, via Ugo La Malfa 153, Palermo 90146, Italy

**Keywords:** biomineralization, transcriptional regulation, signalling, development, ciliary band, *in silico* analysis

## Abstract

Carbonic anhydrases (CANs) are conserved metalloenzymes catalysing the reversible hydration of carbon dioxide into protons and bicarbonate, with important roles in cells physiology. Some CAN-coding genes were found in sea urchin genome, although only one involved in embryonic skeletogenesis was described in *Paracentrotus lividus*. Here, we investigated gene expression patterns of *P. lividus* embryos cultured in the presence of acetazolamide (AZ), a CAN inhibitor, to combine morphological defects with their molecular underpinning. CAN inhibition blocked skeletogenesis, affected the spatial/temporal expression of some biomineralization-related genes, inhibited embryos swimming. A comparative analysis on the expression of 127 genes in control and 3 h/24 h AZ-treated embryos, using NanoString technology, showed the differential expression of genes encoding for structural/regulatory proteins, with different embryonic roles: biomineralization, transcriptional regulation, signalling, development and defence response. The study of the differentially expressed genes and the signalling pathways affected, besides *in silico* analyses and a speculative ‘interactomic model’, leads to predicting the presence of various CAN isoforms, possibly involved in different physiological processes/activities in sea urchin embryo, and their potential target genes/proteins. Our findings provide new valuable molecular data for further studies in several biological fields: developmental biology (biomineralization, axes patterning), cell differentiation (neural development) and drug toxicology (AZ effects on embryos/tissues).

## Graphical abstract

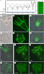

## 1. Introduction

Carbonic anhydrases (CANs) belong to a superfamily of zinc metalloenzymes, including six families (i.e. the α-, β-, γ-, δ-, ζ-, η-CANs), which are widely distributed among living organisms, from plants to vertebrate and invertebrate animals [1]. These enzymes catalyse the reversible hydration of carbon dioxide (CO_2_) to protons (H^+^) and bicarbonate (${\text{HCO}}_{3}^{-}$), and are involved in several crucial physiological processes, including pH and CO_2_ homeostasis/sensing, bone resorption and calcification, respiration and osmoregulation, biosynthetic reactions and many pathological processes [1]. CANs are highly studied since interfering with their enzymatic activity (both inhibition and activation) may lead to pharmacological responses, with potential clinical applications for a variety of disorders [1]. Among the six families, the α-CAN are the most studied and include numerous isoforms showing diverse subcellular localizations, i.e. cytosolic, transmembrane or extracellular [2]. Members of the α-CANs have been found occluded in the extracellular organic matrix of the biomineral structure in some coral, molluscan and sea urchin species, and thus have most often been associated with a variety of roles in skeleton formation (i.e. as a provider of ${\text{HCO}}_{3}^{-}$ ions, a structural protein and a nucleation activator [3]).

In the sea urchin, of the 10 different CAN/CAN-like-coding genes reported in the Echinobase website (www.echinobase.org) [4], the Echinoderm genomic database and the NCBI database, those expressed in the embryonic primary mesenchyme cells (PMCs) are the only isoforms so far described in different sea urchin species (e.g. *Heliocidaris tuberculata* and *H. erythrogramma* [5], *Strongylocentrotus purpuratus* [6,7] and *Paracentrotus lividus* [8]), which had been related only to the biomineralization process.

In our previous study, we have characterized a CAN isoform from *P. lividus* (*Pl*-CAN), which is an α-type CAN with the potentiality to be secreted in the extracellular space, possibly where deposition of the mineralized spicules occurs [8]. Based on *in silico* analysis of *Pl*-CAN sequence, a dual role for the enzyme was envisaged (i.e. as an enzyme regulating ion transport and intra-/extracellular pH and a co-enzyme/regulator of other biomineralization proteins). A specific role in embryonic calcification had been shown in *P. lividus* and *H. tuberculata*, as well as in *Hemicentrotus pulcherrimus* and *Anthocidaris crassispina*, by treatments with a specific CAN inhibitor, acetazolamide (AZ). Indeed, *in vitro* cultures of skeletogenic precursor cells [9] or whole-embryo cultures [9,10], treated with AZ, showed impairment of skeleton formation or elongation. In particular, we have shown that the inhibition of CAN activity completely abolished spicule formation in *P. lividus* embryo, although the up- and downstream cellular and molecular mechanisms related to CAN activity were still largely unknown [10].

Very recently two members of the CAN family have been characterized in *S. purpuratus* PMCs, namely *Sp-Cara2* that is cytosolic, while *Sp-Cara7* displays features of an extracellular enzyme [7]. The knockdown of *Sp-Cara7* by specific morpholino did not completely inhibit spicule deposition but impaired the formation of specific segments of the embryonic skeleton [7]. The sea urchin skeleton formation is a well-documented, complex and multi-step biomineralization process. The embryonic skeleton is produced by the PMCs that form continuous syncytial cables inside which, at the gastrula stage, they begin to deposit the skeletal biomineral in the form of triradiate spicules, which will then elongate according to a species-specific pattern [11]. Several studies, including structural, proteomic and genomic approaches, have addressed the sea urchin skeletogenesis, revealing that it is regulated by a network of regulatory genes/proteins (transcription factors (TFs); growth factors) and the differential expression of their targets (biomineralization proteins; signalling pathways) [12]. The embryonic endoskeleton is composed of calcium carbonate mineral and a soluble organic matrix, composed of a range of macromolecules (more than 200 proteins) identified in *S. purpuratus* by proteomic analysis [13]. To generate the calcitic endoskeleton, PMCs require Ca^2+^ ions as well as dissolved inorganic carbon (DIC, e.g. CO_2_, ${\text{HCO}}_{3}^{-}$ and ${\text{CO}}_{3}^{2-}$). The current knowledge reports that Ca^2+^ ions are acquired from the blastocoel fluid through at least two different pathways: via L-type calcium channels, as assessed using calcium channel inhibitors, and via a passive transport into large networks of vesicles and vacuoles involved in direct seawater uptake [14]. However, the genes/proteins responsible for the specific regulation of Ca^2+^ ions in the PMCs vesicles still remain to be identified. On the contrary, DIC was suggested long ago to derive in part from metabolic CO_2_ and in part from the seawater [15]. A bicarbonate transporter of the SLC4 family has been shown to be critically involved in the PMCs accumulation of ${\text{HCO}}_{3}^{-}$ from seawater, which is required for both intracellular pH (pH_i_) regulation and biomineralization [16]. Recently, immunofluorescence (IF) and single-cell transcriptomic analyses showed a colocalization in the same population of PMCs of the newly identified extracellular *Sp-Cara7* and the *Sp-Slc4a10* [7].The enzymatic activity of CAN as a source of DIC has been reported in other invertebrates. In the freshwater common pond snail *Lymnaea stagnalis*, hydration of CO_2_ catalysed by CAN provides both an endogenous source of ${\text{HCO}}_{3}^{-}$ for calcification and the hydrogen ions to fuel voltage-dependent channels/exchangers for the Ca^2+^ uptake [17]. Calcareous sponges build calcium carbonate spicules through an enzymatic mechanism mediated by a CAN [18], as well as several coral species, as assessed by both pharmacological and histochemical approaches [19].

In addition to the CANs engaged in biomineralization, it cannot be rule out that the other potential isoforms, not yet described in the sea urchin embryo, are involved in other equally vital processes for the organism. Recently, single-cell transcriptomic analyses in *S. purpuratus* pluteus larva detected the expression of some of the CANs found in the sea urchin genome in various cell types, including gut, ectoderm, pigment and muscle cells, neurons [7].

In this study, we used traditional and high-resolution molecular methods to explore gene expression changes in response to treatments of embryos with AZ, which is a specific inhibitor of CAN known to indiscriminately inhibit most of the human isoforms [1], in an effort to combine the morphological defects observed with their molecular underpinning and to possibly figure out the molecular mechanisms correlated with enzymatic activities of different CANs during sea urchin development.

## 2. Material and methods

### 2.1. Collection and embryo culture of *Paracentrotus lividus*

Gametes were collected from gonads of the sea urchin *P. lividus* harvested from the North-Western coast of Sicily (Mediterranean Sea) by local fishermen. Eggs were fertilized and embryos reared at 18°C in artificial Millipore filtered seawater (aMFSW) containing antibiotics (50 mg l^−1^ streptomycin sulfate and 30 mg l^−1^ penicillin), at the dilution of 4000 ml^−1^, with gentle stirring.

### 2.2. Acetazolamide treatment

**Figure 1.**
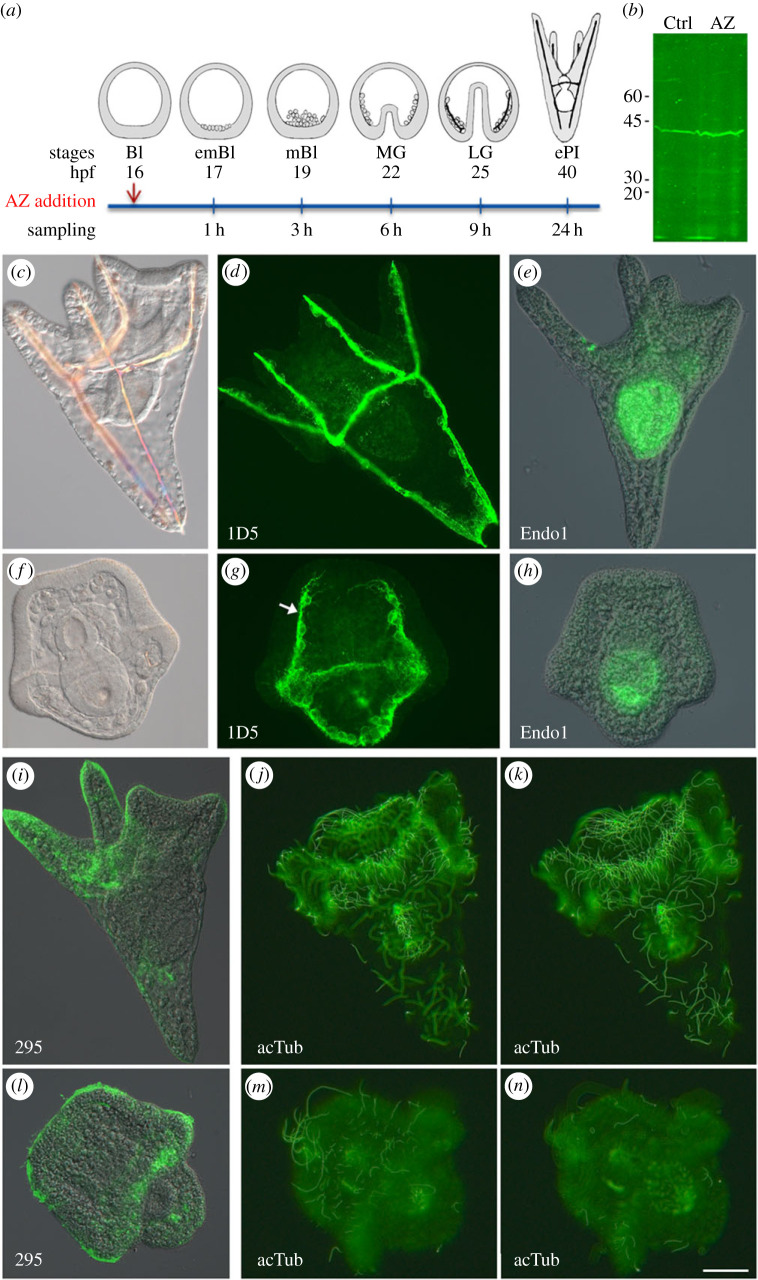
Inhibition of CAN activity by AZ treatment on sea urchin embryo development. (*a*) Schematic drawing depicting the experimental procedure. Blue line, culture in the presence of AZ; red arrow, AZ addition; sampling, sampling times after AZ addition. AZ has been added to blastula embryos (16 h post-fertilization, hpf). Bl, blastula; emBl, early mesenchyme blastula; mBl, mesenchyme blastula; MG, middle gastrula; LG, late gastrula; ePl, early pluteus. (*b*) WB analysis of total proteins from control (Ctrl) and 24 h AZ-treated embryos, reacted with polyclonal antibodies against the recombinant *Pl*-CAN fusion protein. Molecular markers are indicated on the left. (*c*–*e*), (*i*–*k*) Control embryos cultured in the presence of DMSO for 24 h. (*f*–*h*), (*l*–*n*) Embryos cultured in the presence of 8 mM AZ for 24 h. (*d*–*e*), (*g*–*h*), (*i*–*n*) Indirect IF of embryos labelled with 1D5 (*d*,*g*), Endo1 (*e*,*h*), 295 (*i*,*l*), anti-acTub (*j*,*k*,*m*,*n*) antibodies. (*j*,*k*) and (*m*,*n*) are focal planes of the same control and AZ embryo, respectively. White arrow, pseudopodial cables formed by the PMCs. Scale bar, 20 µm.

Acetazolamide (Sigma) was dissolved in dimethylsulfoxide (DMSO) and drug treatments were carried out using the optimal concentration of 8 mM as described by [10]. Briefly, embryos were continuously exposed to AZ from the blastula stage (16 h post-fertilization) until the pluteus (40 hpf) stage (figure 1*a*). Control embryos (Ctrl) were cultured in the presence of the same amount of DMSO as AZ-treated embryos. Embryos were collected at different time points and stored differently for subsequent analyses on the localization and expression levels of both proteins and mRNAs.

### 2.3. Indirect immunofluorescence

IF experiments on whole-mount embryos were performed as previously described [10], with some modifications. Paraformaldehyde-fixed (PFA, Sigma-Aldrich, St. Louis, MO, USA) embryos were blocked with TBST [10 mM Tris-HCl (pH 8), 150 mM NaCl, 0.1% Tween 20] containing 0.5% BSA (BSA/TBST) for 30 min. PMCs, midgut/hindgut and ciliary band (CB) were specifically labelled with monoclonal antibodies 1D5, Endo1 and 295, respectively (a kind gift from Prof. D.R. McClay), diluted 1 : 5. Mouse monoclonal anti-acetylated *α* Tubulin (acTub, Santa Cruz) was diluted 1 : 50. All the primary antibodies were incubated overnight at 4°C with specimens. Secondary antibody Alexa Fluor 488-conjugated rabbit anti-mouse (1 : 200, Invitrogen Molecular Probes, Carlsbad, CA, USA) was incubated for 1 h at room temperature. Labelled embryos were observed under an Axioskop 2 plus microscope (Zeiss, Jena, Germany) and images were recorded by a digital camera system. Images were edited using Adobe Photoshop CS2 software.

### 2.4. Whole-mount *in situ* hybridization

Whole-mount *in situ* hybridization (WMISH) was performed on Ctrl and AZ-treated embryos, fixed in 4% paraformaldehyde (PFA, Sigma-Aldrich, St. Louis, MO, USA) and stored in 100% Methanol at −20°C, as previously described by [20], with some modifications. Hybridizations were carried out with 0.1–1 µg ml^−1^ single-strand sense or antisense DIG- labelled RNA probes at 60°C for 18 h for *sm30* and 42 h for *tbr*, or following the conditions previously reported, i.e. *p16* and *p19* [21], *jun* and *msp130* [20], *sm50* [22] and *can* [8]. After washings, embryos were mounted on glass slides and observed using a Zeiss Axioskop 2 plus microscope; images were recorded by a digital camera. Hybridization with sense probe showed no specific signal.

### 2.5. RNA isolation and real-time quantitative PCR

Total RNA from *P. lividus* control and AZ-treated embryos was reverse transcribed with high-capacity cDNA reverse transcription kit (Applied Biosystems, Grand Island, NY, USA) to obtain the corresponding cDNA as described in Russo *et al*. [23]. In order to quantify gene expression, the cDNAs were amplified by using SYBR Green technology, based on a Comparative Threshold Cycle Method [24], according to the manufacturer's instructions (Applied Biosystems StepOnePlus instrument) and as previously described [23]. *Pl-Z12-1* mRNA was used as internal endogenous reference gene [21]. The primer sequences used for quantitative PCR (qPCR) were previously used: *Pl-alx1* and *Pl-tbr* [22], *Pl-fra1* [23], *Pl-jun* [20], *Pl-foxO* [25], *Pl-pks1* and *Pl-gcm* [26]. The qPCR was run as reported by Chiaramonte *et al*. [22]. Expression levels of the analysed genes are shown as fold change, compared to control embryos developed in DMSO/aMFSW without inhibitor, assumed as 1.

### 2.6. Nanostring nCounter gene expression assay

The differential expression of genes was measured by the nCounter NanoString technology (Diatech Labline) [27], as previously described in Bonaventura *et al*. [28,29]. Briefly, a panel of 127 *P. lividus* transcript sequences were selected and retrieved from the National Centre for Biotechnology Information (NCBI) database (http://NCBI.nlm.nih.gov), including *Pl*-Z12-1 internal reference gene (NCBI, Acc. Number: LT900344). The custom-made probes were hybridized on total RNA (100 ng/sample), extracted from control and AZ-treated embryos using the RNeasy mini Kit (Qiagen, Germantown, MD, USA). The resulting digital counts of each transcript were first normalized using the positive control lane normalization provided in the NanoString nCounter Cartridge and, then, compared between control and AZ-treated embryos to obtain the fold values for each time of AZ treatment analysed (electronic supplementary material, table S1). Values of digital counts less than 100 for controls were considered unreliable and were indicated as ND (Not Determined) in the fold column in electronic supplementary material, table S1.

### 2.7. Western blot analysis

To detect CAN protein, pellets of equal number of control (Ctrl) and AZ-treated embryos were dissociated and lysed in calcium-magnesium free seawater, carefully pipetting embryos up and down to completely lyse them. After vortexing for a few seconds, proteins were precipitated with acetone (3 vol) on ice, o.n. at 4°C. Samples were then centrifuged at 13.000 rpm for 20 min., pellets were left to dry and resuspended with Laemli sample buffer containing β-mercaptoethanol. Proteins were separated by electrophoresis under reducing conditions on 10% SDS-PAGE gel.

To detect phospho-p38-MAPK (p-p38), phospho-p44/42 MAPK (p-Erk) and c-jun, control (Ctrl) and AZ-treated embryos were Dounce-homogenized on ice in Cell Lysis Buffer (Cell Signaling Technology), supplemented with a cocktail of protease (Roche Applied Science, Penzberg, Germany) and phosphatase (Sigma, Chemical Co., St Louis, MO, USA) inhibitors. Proteins were separated by electrophoresis under reducing conditions on 4−15% precast gels (Bio-Rad, Hercules, California, USA).

Western blot (WB) analysis was performed transferring proteins to nitrocellulose membranes using a Semi-Dry electrophoretic transfer cell (Bio-Rad, Hercules, California, USA). Membranes were incubated with Odyssey blocking buffer (LI-COR Biosciences, Lincoln, NE, USA) and then incubated with polyclonal antibodies directed against *Pl*-CAN (1 : 1000; [8]), p-p38 (1 : 250; Cell Signaling Technology Cat# 9211, RRID:AB_331641), p-ERK (1 : 250; Cell Signaling Technology Cat# 9101, RRID:AB_331646), c-jun (1 : 500; Aviva Systems Biology, Cat# ARP30926_P050), α-tubulin (1 : 500; Sigma-Aldrich Cat# T8203, RRID:AB_1841230). The secondary antibodies IRDye 680RD goat anti-rabbit (1 : 5000, LI-COR Biosciences Cat# 926-68071, RRID:AB_10956166) and IRDye 800CW goat anti-mouse (1 : 5000, LI-COR Biosciences Cat# 926-32210, RRID:AB_621842) were incubated for 1 h at room temperature. Proteins were visualized using an Odyssey Infra-red Imaging System (LI-COR) in accordance with the manufacturer's instructions.

### 2.8. Phylogenetic analysis of carbonic anhydrase proteins

The amino acid sequences of CANs from the *S. purpuratus* sea urchin species were obtained from the Echinobase website (www.echinobase.org) [4] and the Echinoderm genomic database (see electronic supplementary material, table S2), while the amino acid sequences of the 14 CANs from the *Homo sapiens* family were obtained from NCBI. All the accession numbers are indicated in figure 7. Multiple alignment of the obtained proteins were performed by MEGAX [30]. Phylogenetic analysis was conducted as previously reported [31], with the only difference concerning the new version of software used, the MEGAX. Multiple alignments of the obtained proteins were performed by Clustal Omega (https://www.ebi.ac.uk/Tools/msa/clustalo/).

### 2.9. Interactomic analysis

The protein–protein interactions were obtained from STRING database (http://string-db.org/, last accessed 14 June 2022). We used *Homo sapiens* as the reference organism because members of CAN families were studied and extensively characterized in *H. sapiens*, while adequate studies are missing for the sea urchin. Moreover, *P. lividus* genes are not even annotated in the STRING database. However, considering the similarity levels of *can* genes between human and sea urchin, we are quite confident that the results obtained are significant and can be easily extrapolated for the sea urchin. In detail, we performed our search as ‘multiple proteins’ mode in different steps, as described in the following. Electronic supplementary material, table S3 shows all the *P. lividus* differentially expressed genes (DEGs, including also p38, ERK and pi3k-110 enzymes) and the corresponding human orthologous genes (protein names and NCBI gene ID numbers) used for the STRING analyses, excluding HE (Hatching Enzyme), MSP130, P19 and SM30 that do not exist in the human database. The network analysis was performed taking into consideration all the seven ‘evidence channels’, i.e. the active interaction sources, including known (databases, experiments) and predicted (gene neighbourhood, fusion and co-occurrence) interactions, as well as text mining (proteins that are frequently mentioned together), co-expression and protein homology, setting the interaction score at ‘low confidence’ (0.150) to expand the number of predicted interactions, although there may be a risk that false-positive or poorly substantiated interactions have also been highlighted. First of all, we searched for the interactions among all the known human proteins coded by the DEGs analysed in this study. In the graphic image, all the proteins were grouped according to the categories described in the text for the *P. lividus* genes. The second step concerned the search of all the known human CAs (a total of 15, electronic supplementary material, table S4) [2]) and the identification of the interactions among each single of them with each group of DEGs. In figure 8, we have not shown every single interaction of the various *hs*CAs (which are however listed in the electronic supplementary material, table S3), but rather we preferred to show the lists of CAs close to each group of DEGs they interact with (identified with the same background colour).

### 2.10. Statistical analysis

QPCR values are reported as the mean of three independent qPCR analyses ± s.d., using different cDNAs obtained by three different experiments of *P. lividus* embryos treated with AZ. Results were compared using Student's *t*-test function on Microsoft Excel.

For WB, fluorescence band values were normalized using α-tubulin and compared to the respective control value, assumed as 1, and are reported as the mean of two–three independent WB analyses ± s.d.

## 3. Results

### 3.1. Inhibition of carbonic anhydrase activity blocks skeletogenesis and swimming of *Paracentrotus lividus* embryos

The observation that AZ, a specific CAN inhibitor, completely prevented the skeleton development [10], provided an opportunity to deepen the study of sea urchin embryo biomineralization. In figure 1*a*, a schematic drawing depicts the timeline of the experimental procedure. In this study, we used the concentration of 8 mM AZ, since it was the minimum dose required to completely inhibit skeleton deposition in *P. lividus* embryos, without affecting the organization of other tissues, compared to control embryos cultured in the presence of DMSO [10]. First, to verify that in AZ-treated embryos the effect was not due to the absence of the protein, we performed WB experiments using polyclonal antibodies against the recombinant *Pl*-CAN fusion protein previously obtained in our laboratory [8]. The antibody detected a band of the expected molecular weight in both control (Ctrl) and embryos treated with AZ for 24 h (24 h-AZ in the following) (figure 1*b*). Unfortunately, the antibody was not useful for assessing the spatial distribution of *Pl*-CAN because it does not work in indirect IF experiments. Focusing on the morphological effects of AZ, we first confirmed the complete absence of the skeleton (figure 1*f* compared to the control in figure 1*c*) in 24 h-AZ embryos, despite the presence of extensive pseudopodial cables formed by the PMCs (1D5; see white arrow in figure 1*g*), correlated with the lack or poorly developed arms. Conversely, a normal development of the tripartite gut was observed, as shown in the DIC image (figure 1*f*) and by the labelling with a hind- and midgut-specific marker (Endo1; figure 1*h* compared to figure 1*e*), as well as the regular localization of a specific marker of the CB (295, figure 1*l* compared to the control in figure 1*i*). An abnormality observed in the behaviour of AZ-treated embryos is noteworthy, as they did not swim during the treatment and remained stationary at the bottom of the well weakly shaking, unlike their controls who usually swim actively in the culture medium in all directions. To deepen this observation, we performed IF experiments using an anti-acTub antibody, specific for the post-translationally modified form of α-tubulin, used as a neural marker labelling neuritis [32], and a marker of the sea urchin cilia too [33]. Ctrl embryos showed many long cilia, mainly located along the CB region (figure 1*j,k*, two focal planes of the same embryo), whereas cilia appeared shorter and in a reduced number in 24 h-AZ embryos (figure 1*m*,*n*, two focal planes of the same embryo).

### 3.2. Inhibition of carbonic anhydrase activity affects spatial and temporal expression of biomineralization- and pigment-related genes

**Figure 2.**
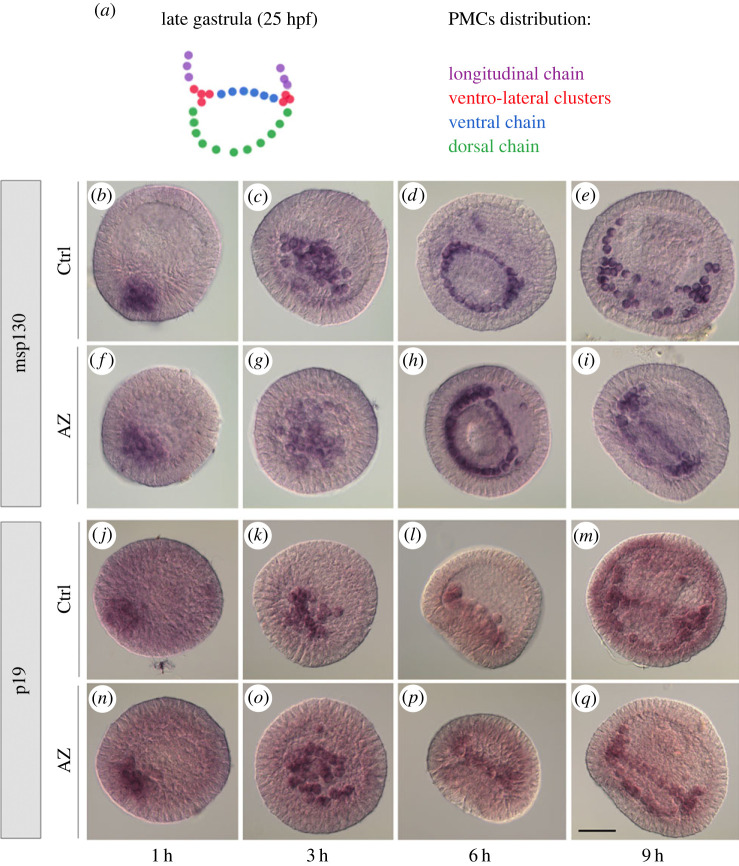
Inhibition of CAN activity does not affect spatial expression of *msp130* and *p19* genes. (*a*) Schematic drawing depicting the PMCs distribution in late gastrula embryo. WMISH of control (Ctrl; (*b*–*e*, *j*–*m*)) and AZ-treated (AZ; (*f–i*, *n*–*q*)) embryos at 1 h, 3 h, 6 h and 9 h with *msp130* (*b*–*i*) and *p19* (*j*–*q*) RNA probes. Scale bar, 20 µm.

We previously [10] showed that, although the major effect of AZ treatment on *P. lividus* embryos was the inhibition of their skeleton development, PMCs migration and differentiation were not impaired. Here, to gain molecular-level information about the AZ treatment, we examined the expression of two mRNAs coding for PMC-specific markers, i.e. msp130, a membrane glycoprotein, and p19, a cytoplasmic protein, from 1 to 9 h after AZ addition (figure 2). Both mRNAs appeared regularly expressed from their first appearance in the presumptive PMCs at the vegetal plate of early blastula embryos (figure 2*f* versus figure 2*b* and figure 2*n* versus figure 2*j*). Intense signal for both mRNAs was apparent in all PMCs throughout the subsequent development of AZ-embryos to the late gastrula stage (figure 2*g–i*, *o*–*q*), as in control embryos (figure 2*c*–*e*, *k*–*m*). These WMISH experiments also confirmed that the spatial and temporal arrangement of PMCs appeared nearly regular within the first hours (from 1 h up to 9 h) of AZ treatment, i.e. until the late gastrula stage (graphically represented in figure 2*a*).

**Figure 3.**
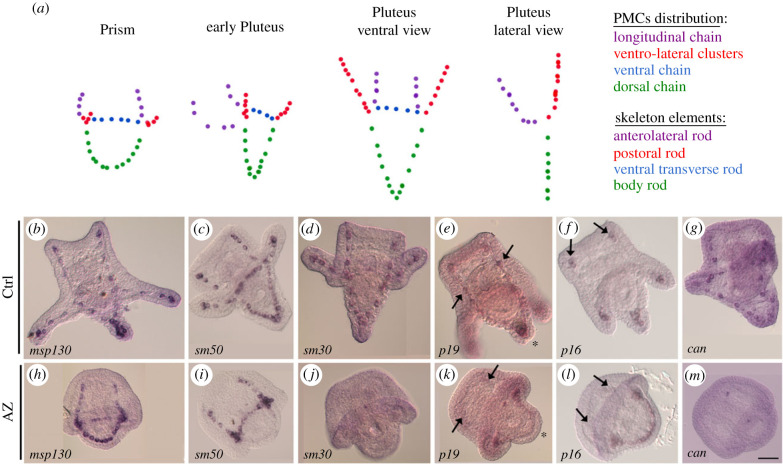
Inhibition of CAN activity affects the spatial expression of biomineralization-related genes. (*a*) Schematic drawing depicting the PMCs distribution and corresponding skeleton elements in Prism, early pluteus and pluteus (ventral and lateral views) embryos. WMISH of control (Ctrl; (*b*–*g*)) and AZ-treated (*h*–*m*) embryos at 24 h with *msp130* (*b*,*h*), *sm50* (*c*,*i*), *sm30* (*d*,*j*), *p19* (*e*,*k*), *p16* (*f*,*l*), *can* (*g*,*m*) RNA probes. Scale bar, 20 µm.

The complete absence of the skeleton should imply that one or more of its protein components were not synthesized, therefore the corresponding mRNAs may not be actively expressed. Therefore, we examined the expression of a number of mRNAs coding for biomineralization-related proteins in 24 h-AZ embryos (figure 3). For reasons of clarity, figure 3*a* shows schematic drawings depicting a timeline of *P. lividus* skeleton development, with the PMCs distribution in each skeleton element synthesized. The *Pl-msp130* (figure 3*b*,*h*) and *Pl-sm50* (figure 3*c*,*i*) mRNAs were regularly expressed in almost all PMCs both in control and in AZ-treated embryos, while *Pl-sm30* mRNA was definitely not expressed in 24 h-AZ embryos (figure 3*j*). Concerning *Pl-p19* and *Pl-p16*, both mRNAs were expressed in some of the PMCs normally expressing them, but not in all. In particular, *Pl-p19* was expressed only in those PMCs localized at the ventro-lateral clusters (figure 3*k*), while PMCs at the dorsal and longitudinal chains did not express this mRNA, differently from the control embryos (see asterisks and black arrows in figure 3*k*,*e*, respectively). *Pl-p16* appeared expressed in PMCs of the dorsal chain and ventro-lateral clusters, while PMCs localized at the longitudinal chains were not expressing it, differently from the control embryos (see black arrows in figure 3*l*,*f*, respectively). Surprisingly, *Pl-can* was apparently not expressed in any of the PMCs in 24 h-AZ embryos (compare figure 3*m* with *g*).

**Figure 4.**
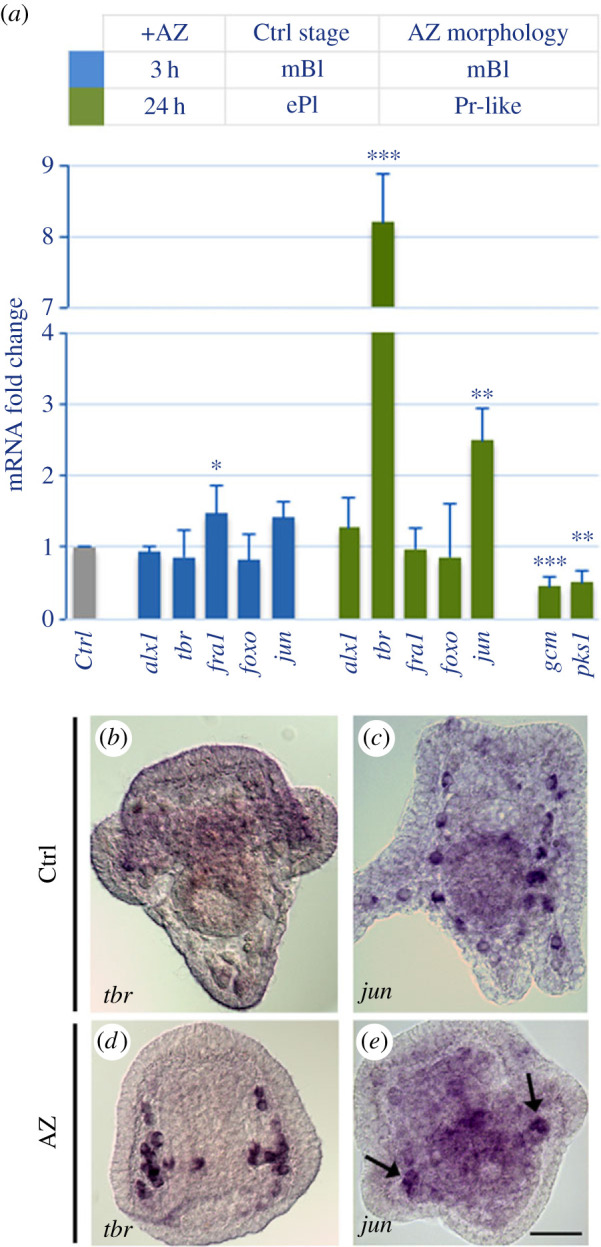
Spatial and temporal expression of biomineralization- and pigment-related genes in AZ-treated embryos. (*a*) Comparative qPCR analyses of mRNA levels in embryos collected at 3 h (blue) and 24 h (green) after AZ addition (+AZ). The stages of the related controls (Ctrl) were mesenchyme blastula (mBl) and early pluteus (ePl), respectively. The morphologies of AZ-treated embryos are also indicated, i.e. mBl and prism-like (Pr-like). Relative mRNA levels are expressed in arbitrary units as mRNA fold change compared to Ctrl samples assumed as one in the histograms, using the endogenous gene *Pl-Z12-1* for normalization. Each bar represents the mean of at least three independent qPCR analyses ± s.d., using cDNAs obtained by three independent AZ-treatment experiments. Mean values were significantly different according to the Student's *t*-test. The asterisks indicate statistically significant variations to the relative control, * (*p* < 0.05), ** (*p* < 0.01, *** (*p* < 0.001). (*b*–*e*) WMISH of control (Ctrl; (*b*,*c*)) and AZ-treated (*d*,*e*) embryos at 24 h with *tbr* (*b*,*d*) and *jun* (*c*,*e*) RNA probes. Scale bar, 20 µm.

We then analysed the expression levels of various mRNAs coding for TFs, known (*alx1* and *tbr*) or supposed (*fra1*, *foxo* and *jun*) to be involved in skeleton development, in 3 h- and 24 h-AZ embryos, by qPCR analysis (figure 4). In general, the expression of all these genes was not significantly affected in 3 h-AZ embryos, while an upregulation was observed for *tbr* (8.2-fold) and *jun* (2.4-fold) in 24 h-AZ embryos (figure 4*a*). To address the spatial expression of these DEGs, i.e. *tbr* and *jun*, WMISH was performed in 24 h-AZ embryos (figure 4*b*–*e*). *Tbr* was regularly expressed in PMCs, with a very intense signal (figure 4*d*) if compared to control embryos (figure 4*b*), that normally at this stage slightly express it, while *jun* was not expressed in all PMCs as in the control embryos (figure 4*c*), but only in those localized to the ventro-lateral clusters (see black arrows in figure 4*e*).

The second important effect caused by AZ was the absence of the red echinochrome pigment in AZ-treated embryos, although the mechanism of action of AZ on pigment cells as well as the functional significance of pigment loss are not yet known [10]. Here, we investigated the expression of the genes coding for polyketide synthase (*pks1*), one of the enzymes involved in echinochrome synthesis, and *gcm*, a TF specific for pigment cells, in 24 h-AZ embryos (i.e. when these cells should have migrated into the larva's ectoderm), and we found that both genes were severely downregulated (about −2-fold) (figure 4*a*).

### 3.3. Inhibition of carbonic anhydrase activity triggers differentially expressed genes

**Figure 5.**
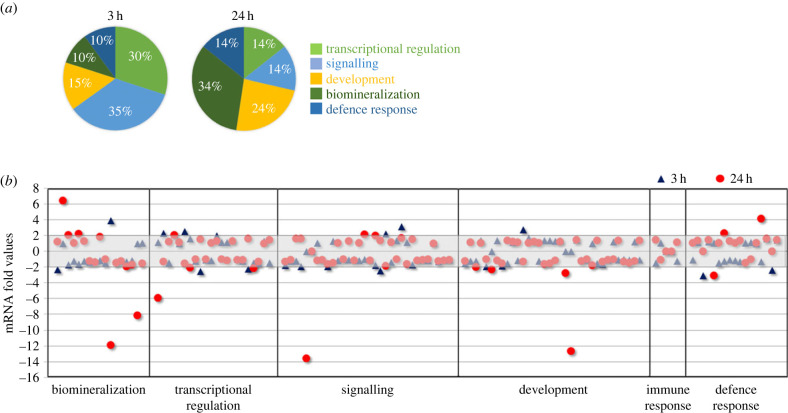
Effects of CAN inhibition on gene expression evaluated by NanoString analysis. (*a*) Graphical representation of genes showing biologically significant fold changes in their expression levels in response to inhibition of CAN activity. Data are presented as the percentage of genes showing significant changes in expression in 3 h and 24 h AZ-treated embryos, compared to control embryos, grouped according to their function (i.e. transcriptional regulation, signalling, development, biomineralization and defence response). (*b*) Scatter graph showing of changes in gene expression levels of 127 genes grouped according to their function (i.e. biomineralization, transcriptional regulation, signalling, development, immune response and defence response). The grey box indicates the threshold for insignificant changes following AZ treatment, i.e. fold values between −2 and +2 at 3 h (blue triangle) and 24 h (red dot).

To deepen our investigation, we used the high-throughput NanoString technology to evaluate the AZ-treatment effects on the expression of 127 selected genes, belonging to different embryonic territories and with different roles during sea urchin development. The analysis was applied on RNAs extracted from control embryos (Ctrl), at the mesenchyme blastula and early pluteus stages, and the corresponding 3 h- and 24 h-AZ embryos. Electronic supplementary material, table S1 lists RAW data and fold changes in the expression levels of the genes analysed, obtained as a ratio between AZ and Ctrl samples. Genes were categorized in six groups by their role or expression site in sea urchin embryo, i.e. biomineralization (including only genes specifically expressed by the PMCs), transcriptional regulation (including various TFs and DNA or RNA binding molecules), signalling (including growth factors and receptors, notch, wnt and hedgehog signalling pathways), development (including animal/vegetal (A/V), dorsal/ventral (D/V) and left/right (L/R) axes patterning, neural differentiation, cell adhesion, cell cycle, metabolism), immune response and defence response (including protein homeostasis, toxicant defence, apoptosis and DNA repair). Focusing on the up- or downregulated genes, the expression levels greater or lower than the fold change threshold of ±2 were taken into consideration, although values greater/lower than ±1.8 were considered biologically significant too. Of the 127 genes quantified using NanoString technology, only 20 (15.7%) and 21 (16.5%) genes were DEG in response to CAN inhibition in 3 h- and 24 h-AZ embryos, respectively. Excluding the genes of the immune response group whose expression did not change following AZ treatment, DEGs were found in all groups analysed. In particular, genes involved in transcriptional regulation and signalling accounted for almost 65% of DEGs in 3 h-AZ embryos (30 and 35%, respectively), while development, biomineralization and defence response genes accounted for 15%, 10% and 10%, respectively (figure 5*a*). Differently, transcriptional regulation and signalling genes accounted for no more than 28% of DEGs in 24 h-AZ embryos (14% each), whereas development, biomineralization and defence response genes accounted for 24%, 34% and 14%, respectively (figure 5*a*). Examining the scatter graph including all the 127 genes, most of the changes in gene expression levels differed slightly from the cut-off values (just outside the light grey area in figure 5*b*), whereas very few genes showed considerable fold changes (greater/lower than ±4) in their expression levels, mainly in 24 h-AZ embryos (figure 5*b*).

The NanoString results suggested that, in general, the expression of some genes is somehow related to CAN activity. In the following, we focused on DEGs within each group, i.e. biomineralization, transcriptional regulation, signalling, development and defence response (table 1). In the biomineralization group, few DEGs were observed in 3 h-AZ embryos, i.e. a downregulated TF, *alx1* (−2.3-fold), and the upregulated spicule matrix protein, *sm30* (+3.9-fold). The greatest number of DEGs was observed in 24 h-AZ embryos, regarding genes coding for some spicule matrix proteins, TFs and CAN, largely in agreement with qPCR results (figure 4*a*). In particular, as expected by the WMISH experiments, the gene expression was severely downregulated for *sm30* (−11.9-fold), slightly downregulated (−1.9-fold) for *p19*, whereas *msp130* was surprisingly slightly upregulated (1.8-fold). An upregulation was also observed for the TFs *ske-t*/*tbr*, *ets1* and *jun* (6.4-, 2.1- and 2.2-fold, respectively). Interestingly, the inhibition of CAN activity by AZ caused a decrease in the expression of its own gene (−8.2-fold). Within the transcriptional regulation group, apart from *dri* that was upregulated in both 3 h- and 24 h-AZ embryos (2.2- and 2.1-fold, respectively), the other 8 DEGs were up- (*hox7*, *nk2.2* and *unc4*) or downregulated (*klf2/4*, *tcf*, *hbox12*, *msx* and *p8*) only in one type of AZ-treated embryo. Regarding the signalling group, the greatest number of DEGs was observed in 3 h-AZ embryos, including genes coding for growth factors (*bmp5/8*, *vegf*), TF (*smad1/5/8*) and kinase (*nlk*) involved in various signalling pathways, and some of the wnt family members (*wnt1*, *wnt3*, *wnt6*, *wnt8* and *wnt16*). Except for *wnt3* and *wnt6*, which were upregulated (2.2- and 3.1-fold respectively), all the others were slightly downregulated. In 24 h-AZ embryos, a slight upregulation (2.0- and 2.1-fold) or downregulation (−1.9-fold) were observed for *wnt1*, *nlk* and *wnt3* respectively. Within the development group, *slc6A4/sert* and *sspo*, a serotonin transporter and an extracellular matrix (ECM) protein both involved in neural differentiation, were highly downregulated (−2.8- and −12.7-fold, respectively) in 24 h-AZ embryos, while we were not able to evaluate any changes in their expression in 3 h-AZ embryos, due to the low RAW data for the 3 h-control embryos, thus suggesting that these genes were almost not expressed at this embryonic stage. In the defence response group, upregulation was observed for *p63* (4.1-fold) and *mdrp1* (2.3-fold) and downregulation for *mt* (−3.1-fold) in 24 h-AZ embryos.

**Table 1.** Gene expression changes in 8 mM AZ-treated embryos at different time intervals (3 and 24 h). Data of expression levels were reported as fold differences (fold change; increase or decrease) in the expression levels obtained as a ratio between RAW data of AZ and control embryos developed in DMSO without inhibitor. The fold change threshold of ±2 was considered significant by statistical analysis, but values greater/lower than ±1.8 are shown since they are considered biologically significant. (−), not affected gene.

DEG groups	genes	fold change after AZ addition		role in sea urchin embryo
		3 h	24 h	
biomineralization	*msp130*	—	+1.8	biomineralization
	*sm30*	+3.9	−11.9	"
	*p19*	—	−1.9	"
	*can*	—	−8.2	enzymatic activity
	*alx1*	−2.3	—	transcriptional control
	*ske-t/tbr*	—	+6.4	"
	*ets1*	—	+2.1	"
	*jun*	—	+2.2	"
transcriptional regulation	*unc4*	+2.3	—	"
	*dri*	+2.2	+2.1	"
	*hox7*	+2.5	—	"
	*klf2/4*	−2.5	—	"
	*nk2.2*	+2.0	—	"
	*tcf/lef*	−2.2	—	transcriptional control/axes patterning
	*msx*	—	−2.1	"
	*p8*	—	−2.2	"
signalling	*vegf*	−1.8	—	GF signalling
	*bmp5/8*	−1.9	—	"
	*smad1/5/8*	−1.9	—	"
	*nlk*	—	+2.1	signalling/axes patterning
	*bmp2/4*	−2.0	—	axes patterning
	*admp1*	—	−2.3	"
	*wnt1*	−1.8	+2.0	Wnt signalling
	*wnt16*	−2.5	—	"
	*wnt3*	+2.2	−1.9	"
	*wnt6*	+3.1	—	"
development	*pitx2*	−1.9	—	neural differentiation
	*otp*	+2.7	—	"
	*slc6a4/sert*	—	−2.8	"
	*sspo*	—	−12.7	"
	*sox9/soxE*	—	−2.0	axes patterning
	*galectin-8*	—	−1.8	cell adhesion
defence response	*hsp70-IV*	−3.1	—	stress response
	*mt*	—	−3.1	"
	*mdrp1*	—	+2.3	"
	*p63*	—	+4.1	stress response/apoptosis
	*hatching enzyme*	−2.4	—	protein homeostasis

### 3.4. Inhibition of carbonic anhydrase activity affects signalling pathways

**Figure 6.**
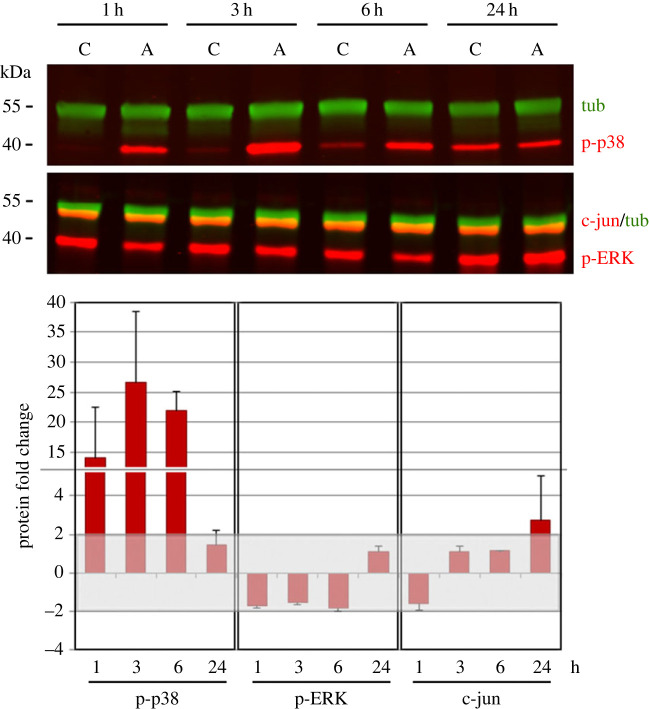
WB analysis of total proteins from control (C) and AZ-treated embryos (A), collected at 1, 3, 6 and 24 h after AZ addition, and probed with anti-p-p38, anti-p-ERK, anti-c-jun and anti-tubulin (tub) antibodies. Molecular weight markers are indicated on the left (kDa). Protein levels represented in the histogram below were calculated as fold increase/decrease of AZ samples compared to controls, normalized using tubulin (tub) detected on the same membranes. Each bar represents the mean value (±s.d.) of at least two independent experiments. Values included within the −2/+2 range (light grey) were not considered as significantly changed.

To also study the signalling pathways potentially involved in CAN activity, we performed WB experiments (figure 6) on total cell lysates from control (C) and AZ-treated (A) embryos, collected at 1, 3, 6 and 24 h after AZ addition, and probed with antibodies against the phosphorylated forms of p38 (p-p38) and ERK (p-ERK), and the non-phosphorylated form of c-jun (figure 6). The protein levels of p-p38 severely increased during the first hours of AZ treatment, i.e. 1 h (13.9-fold), 3 h (26.6-fold) and 6 h (21.9-fold), whereas they did not change in 24 h-AZ embryos. On the contrary, the protein levels of p-ERK did not change significantly, although a slight decrease might be observed during the first hours of AZ treatment, i.e. 1 h (−1.7-fold), 3 h (−1.5-fold) and 6 h (−1.8-fold). The protein levels of jun increased in 24 h-AZ embryos (2.7-fold), although a slight decrease might be observed in 1 h-AZ embryos (−1.5-fold).

### 3.5. Phylogenetic analysis of carbonic anhydrase proteins from *Paracentrotus lividus*, *Strongylocentrotus purpuratus* and *Homo sapiens*

**Figure 7.**
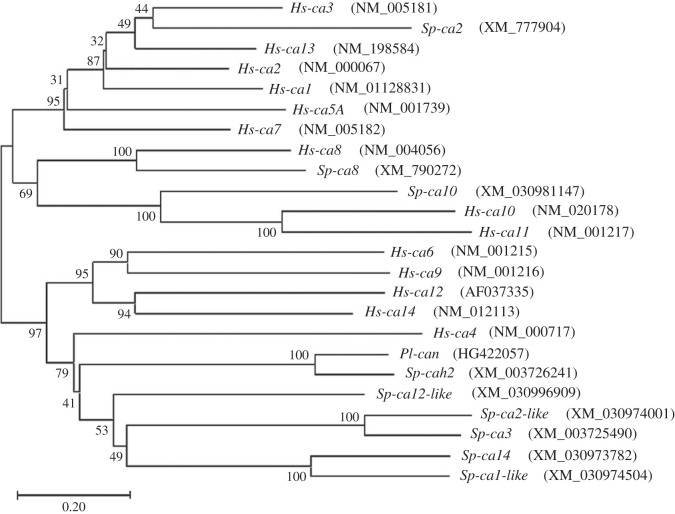
Phylogenetic analysis of sea urchin and human CAN proteins. The phylogenetics was conducted in MEGA X, using the neighbour-joining method. The percentages of replicate trees in the bootstrap test (1000 replicates) are shown next to the branches. The tree is drawn to scale, with branch lengths in the same units as those of the evolutionary distances used. The evolutionary distances were computed using the Poisson correction method and are in the units of the number of amino acid substitutions per site.

On the Echinobase website and the Echinoderm genomic database (www.echinobase.org) [4], 9 genes encoding CAN or CAN-like isoforms (one for each type/isoform, excluding the partial sequences; see electronic supplementary material, table S2) are reported in the *S. purpuratus* genome, although yet to be characterized, but which might be expressed in different cells/tissues of the embryo and have different roles. We compared the only isoform isolated and characterized from *P. lividus* [8] with the CAN/CAN-like isoforms from the *S. purpuratus* sea urchin and 14 CAs from the *H. sapiens* family (one for each type). As expected, the human CA isoforms clustered based on their function and/or location in the cells as shown by the phylogenetic analysis (figure 7), i.e. the clades of cytosolic (*CA3*, *CA13*, *CA2*, *CA1*, *CA5A*, *CA7*) and extracellular (*CA6*, *CA9, CA12*, *CA14*, *CA4*) isoforms, in addition to a separate clade of acatalytic isoforms (*CA8*, *CA10*, *CA11*). Among the *S. purpuratus* isoforms, *Sp-Ca2* (*Cara7LB*), recently shown to be intracellular [7], clustered with *Hs-CA3*, while *Sp-Ca8* and *Sp-Ca10* clustered with *Hs-CA8* and *Hs-CA10* respectively, and all the remaining isoforms clustered together in the clade of human extracellular isoforms, particularly close to *Hs-CA4* (figure 7). *Pl-can* belongs to the extracellular clade, close to *Sp-CAH2* (*Cara7LA*), with 89,2% of similarity, and *Hs-CA4*. The similarity percentages, respect to the number of overlapping amino acids, among *Pl*-CAN and the CAN protein sequences from *S. purpuratus* are reported in electronic supplementary material, table S2, in addition to the list of the synonyms used for the various *Sp*-CAN proteins.

### 3.6. Predicted protein–protein interactions between DEGs/MAPKs and carbonic anhydrases isoforms

**Figure 8.**
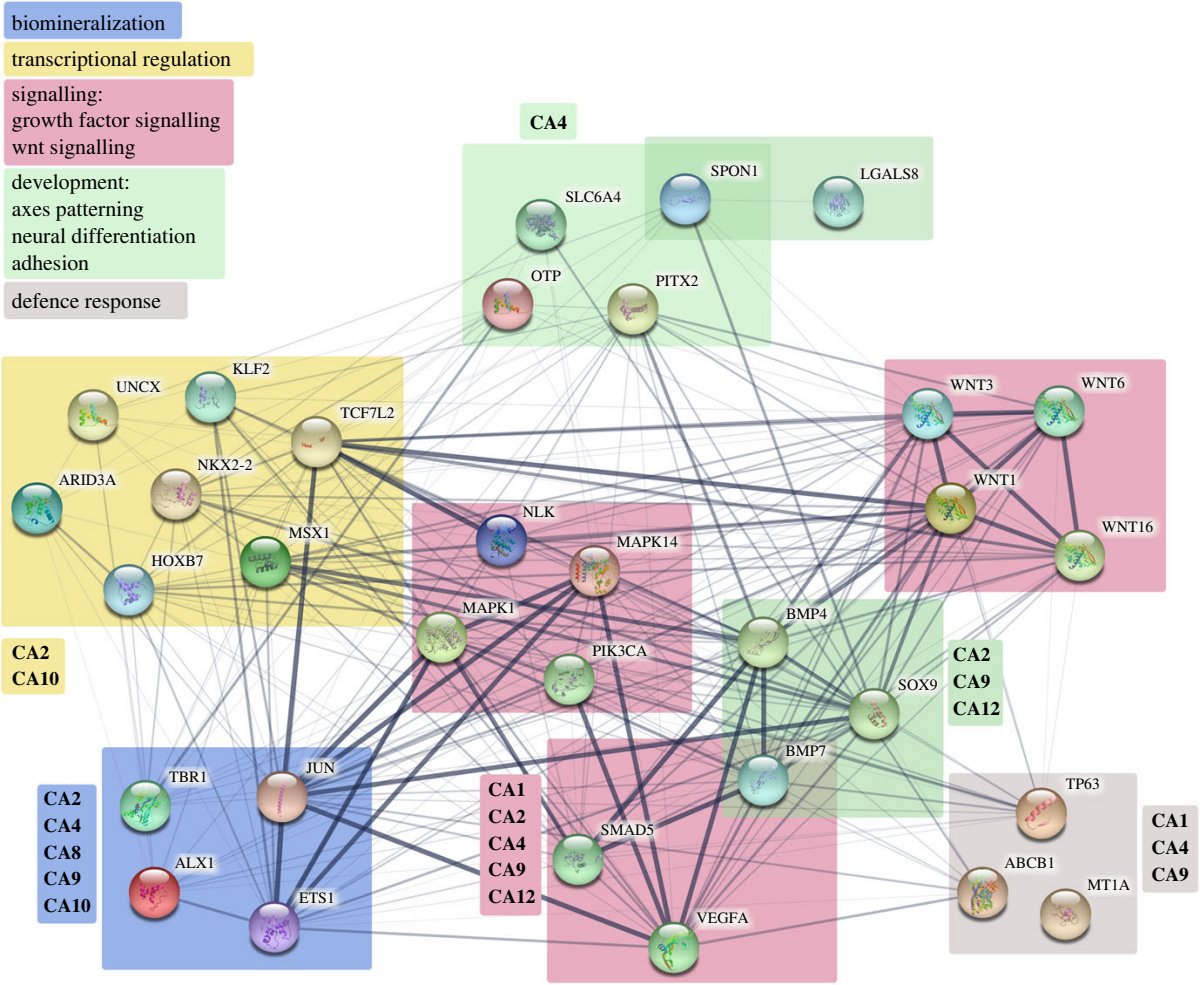
Bioinformatics characterization of the CAN isoforms and DEGs interactome. Human orthologous of the *P. lividus* DEGs were identified and subjected to STRING analysis to reveal interactions among them. The graphic is displayed as nodes, representing proteins and lines, representing the predicted biological relationships between nodes. All the proteins were grouped as described in the text, i.e. biomineralization, transcriptional regulation, signalling (including growth factor and wnt signalling), development (including axes patterning, neural differentiation and adhesion) and defence response. Human CAs are listed close to each group of DEGs they interact with, according to the colour code shown in the legend.

In the attempt to identify those CANs involved in other embryonic processes besides skeletogenesis and to reveal the probable regulators of CAN isoforms or possible targets of their activity in various tissues of the sea urchin embryo, a comparison with molecular data from other chordates can be informative, despite the substantial differences. In particular, we simulated a protein–protein network showing the interactions among all the proteins encoded by the DEGs and the MAPKs analysed in this study, including also PI3K previously studied in our laboratory [22]. We used the STRING database to search for all the predicted connections (physical and functional) among our proteins, setting *Homo sapiens* as the input organism (electronic supplementary material, table S3). Afterwards, after having arranged the proteins according to the DEG groups used in this study (see different colours in figure 8), we looked for all predicted interactions between each group and each human CAN (*Hs-*CA, electronic supplementary material, tables S3 and S4). The interacting *Hs-*CAs were then listed close to each group (without going into the details of the individual interactions for simplicity, see details in the Material and methods section). From a first general analysis, many of the *Hs-*CA isoforms analysed appeared to be connected to the various DEG/MAPK groups, although not all of them, and the prediction showed that some were connected to more than one group and conversely each of the DEG/MAPK groups were connected to more than one *Hs-*CA. In particular, CA2 and CA4 appeared connected to the biomineralization group, the signalling group, particularly to the growth factor and wnt signalling sub-groups, the development group, particularly to the axes patterning, neural differentiation and adhesion sub-groups, while CA2, along with CA10, was also connected to the transcriptional regulation group, and CA4, along with CA1 and CA9, was also connected to the defence response group (figure 8). CA1 also appeared to be connected to the signalling group, whereas CA9 to the biomineralization, signalling and development groups. Finally, CA10 appeared connected to the biomineralization and transcriptional regulation groups, CA12 was connected to the signalling and development groups, CA8 was connected exclusively to the biomineralization group (figure 8).

## 4. Discussion

**Figure 9.**
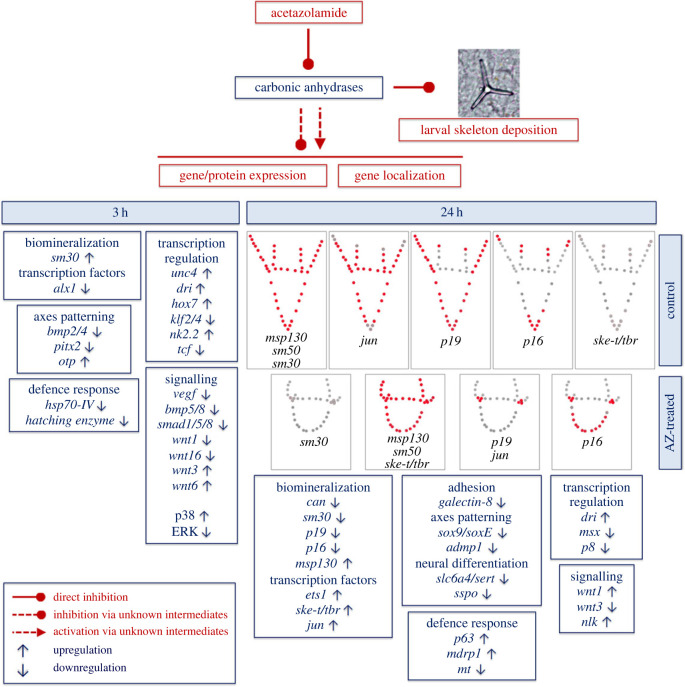
Summary of the genes/proteins expression and localization in 3 h and 24 h AZ-embryos.

In this study, we provide a partial gene expression fingerprint correlated to the sea urchin embryo AZ phenotype, showing the molecular effects of the inhibition of CAN enzymatic activity on the gene expression program responsible for larval skeleton deposition and development, as well as on the expression of some genes coding for molecules involved in growth factors and wnt signalling, axes patterning, neural differentiation and defence response (for a summary of all the results of this study figure 9).

In analysing our data and trying to give them a valid interpretation, it is necessary to address separately two types of results, namely the lack of the skeleton, together with the differential expression of skeleton-related genes, and the changes in the expression of some non-skeletogenic genes, which might be ascribed to the presence of more than one CAN isoform localized in different tissues of the sea urchin embryo. Furthermore, we may assume that inhibition of CAN enzymatic activity was continuous throughout the incubation period, albeit temporary, as we have previously shown that the inhibitory effects of AZ are completely reversed after its removal from the medium, and the skeleton-deficient embryos are able to recover skeletal development [10].

The inhibition of skeleton development raised the main question of how CAN enzyme might be involved in the biomineralization mechanism of the sea urchin embryo. To date, the only information in the *P. lividus* species concerned the expression of a *Pl-can* mRNA in the PMCs, in addition to a generic role in skeleton development validated by pharmacological inhibitor studies [8,10]. From here on, the gene corresponding to the isoform involved in biomineralization will be referred to as *Pl-can*, while the other hypothetical isoforms will be generically indicated as *can*. Considering that its enzymatic activity consists in catalysing the reversible hydration of CO_2_, we assumed that *Pl*-CAN enzyme was responsible for providing the primary substrates for biomineralization, i.e. the ${\text{HCO}}_{3}^{-}$ ions required for the final CaCO_3_ deposition. Indeed, very recently, the extracellular *Sp-Cara7* has been shown to promote a carbon concentration mechanism in *S. purpuratus* PMCs, namely generating ${\text{HCO}}_{3}^{-}$ ions from metabolic CO_2_, collaborating with ${\text{HCO}}_{3}^{-}$ transporters involved in biomineralization [7].

The biomineralization process in marine calcifying organisms usually involves two combined mechanisms, i.e. the transport of ions (Ca^2+^, ${\text{HCO}}_{3}^{-}$ and H^+^) at the mineralization sites, by carrier molecules and/or vesicles-mediated transport, and the synthesis and secretion of macromolecules forming the organic matrix of the skeleton, controlling the crystal nucleation, the amorphous calcium carbonate (ACC) stabilization and the spatial orientation of the growing skeleton [19,34–36]. In sea urchin embryo, the PMCs are responsible for skeleton formation, both synthesizing and secreting spicule matrix proteins and producing the ACC within cytoplasmic vacuoles and vesicles, subsequently exported to the syncytium [37]. Numerous data are available concerning the cellular processes involved in the ACC formation, including the supply of bicarbonate or carbonate ions at sites of biomineralization, the pathways of calcium transport and concentration within cytoplasmic particles/vesicles, the dynamics of vesicle motion and the molecular mechanisms controlling it, the ACC transfer to the syncytium [14,37–39]. Nevertheless, the involvement of *Pl*-CAN activity in the deposition of the skeletogenic biomineral in the sea urchin embryo has not yet been described at the molecular level. Actually, by a biochemical and proteomic approach, a minimal set of proteins directly involved in the mineral deposition has been identified in the test matrix of *P. lividus* adults [8]. *Pl*-CAN was among the 10 occluded proteins identified, which were tightly bound to the mineral phase, and was present in both acetic acid-soluble and -insoluble matrices of the skeletal test tissues. By an *in vitro* crystallization assay, authors showed that this set of proteins has a shaping effect on the biocalcite formation, thus providing new information to the biologically controlled mineralization hypothesis [40]. The presence of *Pl*-CAN in adult tests was confirmed by WB using the same homologous polyclonal antibody used here to verify the presence of *Pl*-CAN in AZ-treated embryos (figure 1*b* this manuscript), indicating that *Pl*-CAN is a component of both embryonic and adult skeletal matrix and thus suggesting that it might have a similar behaviour in the adult and embryonic biomineralization processes. However, it would be important in the future to verify and confirm the predicted extracellular localization of *Pl*-CAN also in the *P. lividus* embryo.

The absence of the skeleton in AZ-treated embryos may be due to some dysfunctions in PMCs or to reduced ${\text{HCO}}_{3}^{-}$ supply. At the same time, changes in the expression of most of the genes analysed here might be a consequence of intracellular pH (pH_i_) alteration, as it is known that pH_i_ disturbances are associated with a wide range of cellular dysfunctions, or the results of more generic changes in metabolism in attempts to minimize CAN enzymatic activity inhibition.

### 4.1. Acetazolamide treatment and skeleton absence

As expected, *Pl*-CAN inhibition did not affect PMCs migration, as the phenotype of AZ-treated embryos was characterized by a regular migratory behaviour and correct arrangement in a typical pattern of PMCs inside the blastocoel ([10]; this manuscript). A similar phenotype in sea urchin embryo has been shown in previous studies using pharmacological approaches [22,41,42]. A number of evidence indicate that *Pl*-CAN inhibition by AZ treatment did not affect PMCs differentiation too, as these cells are able to form pseudopodial cables (labelled by the 1D5 antibody, figure 1*g*), indicating that the fusion process is not prevented, and express some PMC-specific markers as shown by WMISH, i.e. either genes coding for spicule matrix proteins (*msp130* and *sm50*, this manuscript) or TFs (*tbr*, this manuscript; *alx1*, not shown). Nevertheless, although they are regularly expressed even in the absence of *Pl*-CAN activity (3 h- and 24 h-AZ embryos), MSP130 and SM50 are not sufficient to start skeleton synthesis/deposition, maybe requiring additional posttranscriptional activation or the concurrent presence of other biomineralization molecules.

Considering that not even a small spicule rod has ever been observed at the AZ dose used, we can suppose that AZ-treated embryos might be deficient in the fundamental elements required for skeleton deposition within the syncytium. Therefore, by inhibiting *Pl*-CAN enzymatic activity, AZ might prevent or cause insufficient CO_2_ hydration, thus reducing the supply of both H^+^, involved in voltage-dependent channels/exchangers for the ${\text{Ca}}_{2}^{+}$. uptake, and ${\text{HCO}}_{3}^{-}$, required for the final production of CaCO_3_. Alternatively, AZ treatment might indirectly reduce the ${\text{HCO}}_{3}^{-}$ intake. Indeed, it has been reported that different CANs can bind to several acid/base-coupled membrane transporters, thereby increasing their activity. For example, the CAII activity appears necessary to enhance the transport capacity of the chloride/bicarbonate exchanger AE1 in human erythrocytes or of the sodium-bicarbonate co-transporter NBC1 in *Xenopus laevis* oocytes [43,44]. In both cases, pharmacological inhibition of CAII by specific inhibitors (i.e. AZ or ethoxyzolamide) significantly reduces bicarbonate transport. A functional complex or ‘transport metabolon’, composed of CAII and bicarbonate transporters, has been hypothesized [45], possibly involved in the regulation of bicarbonate metabolism and transport, although a real physical contact between the two molecules is still controversial and is a topic of discussion [44,46,47]. Nevertheless, a number of ion transporters/exchangers has been annotated in the sea urchin genome and some have even been described and characterized in various species and different tissues and with varied functions [16,48,49]. In particular, *Sp*SLC4a10, belonging to the SLC4 bicarbonate transporter family, has been recently identified in PMCs of *S. purpuratus* embryo and its role in biomineralization via pH_i_ regulatory and ${\text{HCO}}_{3}^{-}$ concentration mechanisms has been hypothesized [16]. The colocalization of *Sp-Slc4a10* and *Sp-Cara7* recently shown in the same population of *S. purpuratus* PMCs [7] suggests the presence of ‘transport metabolons’ even in the sea urchin embryo, whose activity might be indirectly inhibited by AZ treatment with a consequent reduced ${\text{HCO}}_{3}^{-}$ intake in PMCs. Obviously, this issue remains highly speculative and needs further investigation with appropriate approaches.

### 4.2. Differentially expressed genes of the biomineralization group

The spicule matrix proteins analysed in this study are known or supposed to be differently involved in skeleton development and to have different localization within it, i.e. on PMCs cell surface (MSP130 and P16) [50,51], in PMCs cytoplasm (P19) [21,52], occluded in the organic matrix (SM50 and SM30) [53] or extracellularly localized (*Pl*-CAN) [8].

Concerning the biomineralization group, only two genes were differentially expressed in 3 h-AZ embryos (*sm30* and *alx1*), whereas 8 out 16 genes were differentially expressed in 24 h-AZ embryos (as shown by NanoString (electronic supplementary material, table S1) and/or WMISH (figure 3) analyses) and of these, four were spicule matrix proteins (*msp130*, *sm30*, *p19 and p16*), one enzyme (*Pl*-*can*) and three were TFs (*ske-t/tbr*, *ets1* and *jun*) (summary in figure 9). The reduced expression of *alx1* in parallel with the increased expression of *sm30* in 3 h-AZ embryos might suggest a negative control of this TF on *sm30* under normal conditions at the mesenchyme blastula stage. It is actually known that the *sm30* gene is not normally expressed at this stage of development, but it starts to be so later, i.e. at late gastrula, when the spicules begin to be deposited [54], and, as far as we know, up to now its expression seems to be under the sole control of VEGF and no potential TF involved has yet been identified [55]. On the contrary, the severe inhibition of *sm30* expression in 24 h-AZ embryos was quite predictable, since it is known that this gene is responsive and/or affected by external cues, both cellular and environmental [22,55–58]. The decrease in *p19* mRNA levels in 24 h-AZ embryos (NanoString analysis) must be related to its downregulation in PMCs at the tips of the anterolateral and body rods (WMISH experiments), whereas the spatial expression of *p16* was downregulated only at the tips of the anterolateral rods, even if its mRNA levels were not noticeably affected (NanoString analysis) (see summary in figure 9). From multiple sources [6], it has been highlighted that different PMCs have distinctive roles according to their position along the growing skeleton rods, suggesting that a dynamic modulation of the functional states in mineralizing PMCs is needed for the formation of an elaborated skeleton. Intriguingly, the spatial expression of *p19* in 24 h-AZ embryos was concentrated in the PMCs localized at the ventro-lateral clusters, similarly to that of *jun* (figure 9), whereas these two genes are expressed in almost all PMCs in normal embryos [20,21]. This might be a fortuitous observation, or it could highlight a cause–effect relationship. That is to say, could the increase in *jun* expression be correlated with the decreased expression of *p19* in the ventro-lateral cluster PMCs? Actually, we only know that the changes in *jun* spatial expression in 24 h-AZ embryos (WMISH, figure 4) is associated with increased levels of both its mRNA (NanoString analysis), which was previously observed as a response to UVB stress [25], and protein (WB analysis, figure 6). To date, a role for *jun* in embryonic skeletogenesis has only been suggested on the basis of its spatial expression during sea urchin development [20]. Further studies on *jun* mode of function are awaited.

Concerning the other DEGs of the biomineralization group, the upregulation observed for *msp130* and the TFs *ske-t/tbr* and *ets1*, by NanoString analysis and WMISH, was unexpected. In general, it seems as if the crystal nucleation failure as a consequence of *Pl*-CAN enzymatic activity inhibition might be sensed by PMCs, which in turn modifies the regular gene expression program responsible for spicule deposition, although at the moment, we cannot envisage whatever mechanism of action.

### 4.3. The inhibition of can gene expression

Many sulphonamide drugs impact the structural properties of the enzyme they act on, as well as AZ occupies the catalytic site of CAN, thus inhibiting its enzymatic activity by a competitive mechanism [59]. As expected, *Pl*-CAN protein levels did not appear to be affected in 24 h-AZ embryos, as shown by the WB analysis. By contrast, the severe inhibition of *Pl*-*can* gene expression (reduced by 8.2-fold compared to controls) was quite surprising and difficult to explain, even though it is known that changes in mRNA levels do not necessarily reflect changes in protein amounts or in enzymatic activity. Many studies have recently described the sensitivity of specific CAN isoforms to environmental changes, in terms of pH, temperature and salinity, in numerous aquatic calcifying invertebrates, which respond, among others, also in terms of variation of *can* gene expression, generally downregulating it as a consequence of pH reduction [60]. Therefore, the reduction in the abundance of *Pl*-*can* mRNA might be the result of changes in the intracellular and/or extracellular conditions caused by the AZ treatment. Nevertheless, it remains important to understand how the inhibition of the enzymatic activity of *Pl*-CAN and other predicted CAN isoforms is transduced at the molecular level, both in modulating the expression of its own gene and that of others.

### 4.4. Disturbance of pH_i_ homeostasis could result in differential expression of genes from various groups

Among the various physiological processes in which it is involved, CAN also plays a role in pH homeostasis. Examples of CANs controlling cell differentiation by regulating pH_i_, and the related effects of their inhibitors, have been reported, such as during osteoclastogenesis and amelogenesis in vertebrates [61,62]. Intracellular pH is known to be a critical factor for any cellular process and its improper variation is associated with a wide range of cellular dysfunctions, affecting many intracellular signalling systems with an ultimate impact on the expression of some genes [62,63]. Therefore, we might suppose that the inhibition of CANs enzymatic activity by AZ treatment, in addition to potentially deprive the PMCs of the fundamental ions required for skeleton deposition, might affect the optimal pH_i_, not only in PMCs but also in other cells/tissues expressing additional CAN isoforms. The presence of various genes encoding CAN/CAN-like isoforms can be hypothesized in the *P. lividus* species, similarly to what is observed in the *S. purpuratus*, where the expression of different *Sp-can* genes (*Sp-Ca2*, *Sp-Ca14*, *Sp-Ca14-like*, *Sp-beta Ca1* and *Sp-Ca10*) was detected in various cell types, including gut, ectoderm, pigment and muscle cells, neurons, by single-cell transcriptomic analyses [7]. In *P. lividus*, we had already hypothesized the presence of at least one CAN isoform in pigment cells, a subpopulation of non-skeletogenic mesodermal cells with immune activities within the larva [10]. In particular, we showed that the AZ-treated embryos were completely devoid of the echinochrome pigment normally produced by pigment cells, thus providing the first evidence of a potential CAN enzymatic activity in larval immune pigment cells. This hypothesis is supported by the severe downregulation of both pigment cells-specific genes, i.e. *gcm* and *pks1*, in 24 h-AZ embryos, as shown by our qPCR analysis (figure 4) and by the expression of *Sp-Ca2* in *S. purpuratus* pigment cells [7]. In agreement, a role for CA14 in zebrafish melanocytes differentiation has been recently established after its silencing, which resulted in the formation of immature acidic melanocytes with reduced pigmentation, together with a downregulation of pigmentation-promoting gene expression [64].

Most of the DEGs of the ‘transcriptional regulation’ group are known to be expressed in dorsal (aboral) and/or ventral (oral) ectoderm cells, although the function of many of them is not yet well known, including *unc4*, *dri*, *hox7*, *nk2.2*, *klf2/4* and *tcf* (upregulated/downregulated in 3 h-AZ embryos), *msx* and *p8* (downregulated in 24 h-AZ embryos), *dri* (upregulated in 3 h- and 24 h-AZ embryos). At the moment, we can only be aware that their function may depend on the proper functioning of CANs.

Within the ‘signalling’ group, the ectoderm-derived VEGF is known to regulate various steps of skeletogenesis and, although its downstream signalling is not yet completely uncovered, to date it is the only acknowledged regulator of *sm30* expression [55]. Interestingly, the inhibition of VEGF signalling by treatment of *L. variegatus* blastula/early gastrula embryos with axitinib, a VEGF receptor (VEGFR) inhibitor [41], leads to phenotypes similar to those obtained after treatment of *P. lividus* blastula embryos with AZ ([10]; this manuscript). Recently, we have shown that the phosphatidylinositide 3-kinase (PI3K) signalling pathway is involved in *P. lividus* skeletogenesis, as also in that case the treatment of *P. lividus* blastula embryos with LY294002 (LY), a PI3K inhibitor, produced embryos completely lacking the skeleton, but with normally differentiated ectoderm and endoderm tissues [22]. In that study, it was noteworthy to underline that all three treatments, i.e. axitinib, LY and AZ, caused the inhibition of *sm30* expression. As a result of *in silico* analyses, we proposed a ‘*Pl*-PI3K interactomic’ model, i.e. a novel regulatory step in the embryonic skeletogenesis, which hypothesizes a direct regulatory connection between PI3K and VEGF, and an indirect link to CAN and SM30, namely PI3K-VEGF-CAN-SM30 sub-circuit [22]. Obviously, direct evidence is needed to verify if PI3K signalling is one of the pathways activated downstream of VEGF to regulate CAN and SM30 functions. Recently, Morgulis *et al*. [65] showed that CAN-like 7 (*Pl-caral7*) is among VEGF targets, in addition to *sm30* and to members of solute carrier ${\text{HCO}}_{3}^{-}$ transporter families (*Pl-slc26a5*), the latter having a role in pH regulation in PMCs.

Although no alteration of the development of the various axes was observed at morphological level after AZ treatment (this study; [10]), some of the genes involved in their specification and patterning (belonging to the ‘signalling’ and ‘development/axis patterning’ groups) were differentially expressed (up- or downregulated) in both 3 h- and 24 h-AZ embryos. It is known that multiple intracellular signals cooperate to establish the various axes, with the contribution of reciprocal signalling between the ectoderm and the endomesoderm and of regulatory factors involved in several positive and/or negative feedback circuits [66–68]. At present, however, the differential expression of each of these genes (i.e. the growth factors BMP5/8, BMP2/4 and ADMP1, the signal transducer SMAD1/5/8, the TFs SOX9/SOXE and TCF/LEF, the kinase NLK, some WNT signalling ligands) in AZ-treated embryos is difficult to explain, but in general our results suggest some involvement of CANs enzymatic activities in their regulation as well as in the formation of the embryonic axes.

The inhibition of CAN activity can also affect the embryonic nervous system of the sea urchin, as suggested by some behavioural and molecular evidence. The inhibition of the embryonic swimming might be related to some type of damage to the CB, which is the main organ controlling swimming and feeding in the sea urchin larva [69]. CB is a well-defined embryonic region localized between oral and aboral ectoderm and represents the sea urchin embryo's peripheral nervous system, being rich in sensory neurons, which respond to environmental stimuli and control ciliary cells beating [70,71]. Actually, AZ-treated embryos still formed a CB, as they expressed the ciliary marker 295, but showed a reduced number of cilia, which were also shorter than those of controls, thus suggesting that the swimming inhibition might be due to some kind of defect in cilia development or to defects in CB neurons activities. Aberrant swimming behaviour has been observed in zebrafish embryos treated with sulphonamides inhibitors, including also AZ, probably induced by damages to CANs physiological functions, including pH maintenance and ion transport [72].

At the molecular level, the CB specification is a complex process, which involves the activity of numerous genes and signals from different neighbouring territories [73]. Among the CB specification genes indicated by Burke *et al*. [74], our NanoString analysis considered *foxG*, *gfi1*, *onecut*, *otx*, *pax2/5/8*, *univin* and *wnt8*, of which two were differentially expressed in our AZ-treated embryos, i.e. a slight downregulation (considered biologically significant even though under the established threshold) was observed for *wnt8* (−1.7 in 3 h-AZ embryos) and *pax 2/5/8* (−1.7 in 24 h-AZ embryos). Furthermore, the expression of two potential neural marker genes, such as *slc6a4/sert*, a serotonin transporter and *sspo*, an ECM protein, appears to be somewhat related to CAN activity at late embryonic development, as suggested by their downregulation in 24 h-AZ embryos. Conversely, the inability to measure their expression in 3 h-control embryos suggested that these genes were almost not expressed at this embryonic stage, i.e. early gastrula stage, 19 hpf. This result is in agreement with RT-PCR analysis performed by Nikishin *et al*. [75], who showed that a *sert-like* gene, despite being expressed in *P. lividus* sea urchin oocyte and after fertilization, was not expressed at blastula and gastrula stages, only to appear again at the late stages (prism and pluteus). Although a serotonergic nervous system has been described in the sea urchin larvae, which appears involved in the regulation of their swimming behaviour [76–78], an involvement of the serotonin transporter slc6a4/sert has not yet been hypothesized. The *sert* gene has been associated with many human behaviours, both normal and pathological, as well as with some neurodevelopmental disorders like autism [79]. On the contrary, there is no information on the expression (neither temporal nor spatial) of *sspo* gene in the sea urchin embryo. This gene codes for a large multi-domain protein, which is probably involved in axonal growth and/or guidance during neural cell differentiation in Chordates [80]. Meiniel *et al*. [81] showed that *sspo* is expressed in the neural tube of the zebrafish embryos and that its multi-domain structure is conserved from Echinodermata to Vertebrata, therefore suggesting a common function in different phyla. Here, we have only shown the existence of a link, albeit indirect, of the CAN activity with the expression of SLC6A4/SERT and SSPO, the meaning of which still remains unknown, and which is worth investigating in the future.

Even the MAPK signalling pathways seem to be related to CAN enzymatic activity, although the possible relationship between the inhibition of the latter and the variations in the levels of the phosphorylated forms of the two MAPKs, p-p38 (increase) and p-ERK (slight decrease) (figure 6), remains to be clarified. A possibility is that, in 1 h- to 6 h-AZ embryos, phosphorylation of p38-MAPK and ERK was, respectively, activated or inhibited because of pH_i_ changes caused by the CAN activity inhibition, which in contrast did not affect *p38* gene expression. It has been shown that p38-MAPK signalling pathway might be activated by extra/intracellular pH changes. For example, a significant pH_i_ decrease in HepG2 cells treated with a cardiovascular hormone was coupled to an increased p38-MAPK phosphorylation, which led to the downregulation of the Na^+^/H^+^ exchanger NHE-1 mRNA and protein levels [82]. In addition to the binding to chloride/bicarbonate exchanger AE1 or sodium-bicarbonate co-transporter NBC1 in vertebrates as discussed above, CAII has been shown to modulate mammalian NHE-1 activity, by a phosphorylation-regulated binding to a specific site at the C-terminal of NHE-1 [83]. The NHE3, together with other ion transporters, has been localized in the stomach epithelium of *S. purpuratus* pluteus, where it appears involved in the gastric pH regulatory machinery [84]. In the hypothetical model to explain gastric alkalization in pluteus larva, the authors also suggested the involvement of CAN activity. Other NHE homologues are present in the *S. purpuratus* genome (at least four genes according to the Echinobase database) and thus a link between one of the CAN isoforms and NHEs, involving MAPKs signalling, might be envisaged in the sea urchin. Phosphorylation is an important post-translational modification event, which requires the presence of specific amino acidic residues within the protein sequence. In hCAIX, the phosphorylation of its Y449 residue is known to lead the enzyme to interact with PI3K kinase [85] and we have already shown a link between PI3K signalling pathway and *can* gene expression in the sea urchin embryo [22].

### 4.5. An ‘interactomic model’ of carbonic anhydrases isoforms

**Figure 10.**
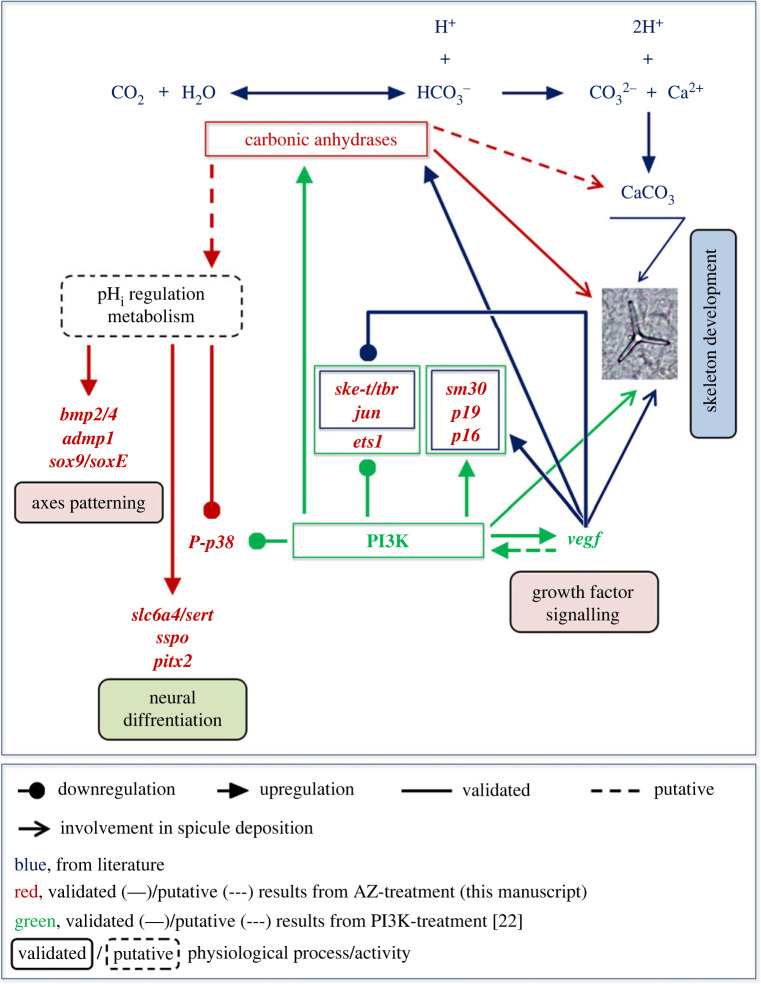
Interactomic model of CAN isoforms in *P. lividus* embryo. The model simulated connections of CANs to different proteins and to physiological processes/activities occurring from the blastula stage onwards, including both putative (dashed lines/arrows) and validated (solid lines/arrows) data from literature (blue), results from AZ-treatment (red, this study) and results from PI3K-treatment (green) [22]. The model does not contain information on the possible subcellular localization of the potential *P. lividus* CAN isoforms.

This is the first study that investigated the effects of inhibiting CAN enzymatic activity on the sea urchin embryo development at the molecular level. Here, we provided new and interesting (albeit somewhat preliminary) data that could be helpful for understanding the role of CANs both in skeletogenesis and in other physiological processes in invertebrate, as well as vertebrate organisms, given that these enzymes are widely expressed and conserved in most species. In the *S. purpuratus* genome, at least nine genes coding for CAN or CAN-like isoforms have been reported, which require to be characterized. Although our study does not clearly identify the various predicted CAN isoforms (which actually was not the purpose), it does provide essential molecular-level insight towards understanding what different processes they might be involved in during the development of the sea urchin embryo. Based on the results shown and the ‘*in silico*’ analysis performed through human STRING database, we have arranged an ‘interactomic model’ (figure 10) to simulate the possible involvement of CAN isoforms in different embryonic processes occurring from the blastula stage onwards, with potential target genes/proteins. The evident involvement of CANs in sea urchin biomineralization, possibly with a role in CaCO_3_ deposition, might occur in association with PI3K signalling, which has been shown to be a novel regulatory pathway for embryonic skeletogenesis [22,86]. Indeed, the expression of some biomineralization-related genes (i.e. *tbr*, *jun*, *ets1*, *sm30*, *p19* and *p16*) might be indirectly regulated by CAN through the pH_i_/metabolism regulation and the PI3K signalling pathway (figure 10). A ‘PI3K-VEGF-CAN-SM30 sub-circuit’ like the one previously suggested [22] could account for *sm30* and *vegf* downregulation when CAN enzymatic activity is inhibited. In agreement, our STRING analysis predicted that both PI3K and VEGFA were interacting partners of some of the cytosolic/membrane-bound *hs*-CA isoforms, i.e. CA9 for both proteins and CA1, CA2, CA4 and CA12 for VEGFA (see electronic supplementary material, table S3).

The ‘interactomic model’ for the first time suggests a role for CANs even in axes patterning and neural differentiation during sea urchin embryo development, probably again through the regulation of pH_i_ or cellular metabolism (figure 10). In particular, our STRING analysis disclosed SOX9 as an interacting partner of the cytosolic CA2 and the transmembrane CA9 and CA12, while SLC6A4 was predicted to be an interacting partner of the GPI-anchored CA4 (see electronic supplementary material, table S3).

Needless to say, the proposed ‘interactomic model’ is not exhaustive, since numerous questions still remain unanswered, as for example the subcellular localization of the different CAN isoforms, and requires further studies and experimental evidence for its validation and improvement.

## 5. Conclusion

Based on currently known literature, it appears clear that the presence of several CAN isoforms, showing different subcellular localizations and functions, is of vital importance for correct embryonic development. In agreement, our results suggest that CANs are involved in skeletogenesis as well as in various homeostatic processes during sea urchin embryo development, and the inhibition of their activity induces morphological and/or behavioural abnormalities by affecting different signalling pathways.

We are confident that the molecular data shown, together with the proposed ‘interactomic model’, could be useful in several biological fields, such as developmental biology (biomineralization and axes patterning studies), cell differentiation (neural development studies) and drug toxicology (studies on AZ effects on embryos/tissues).

## Data Availability

The data are provided in the electronic supplementary material [87].
